# Within-Plant and Within-Field Distribution Patterns of Asian Bean Thrips and Melon Thrips in Snap Bean

**DOI:** 10.3390/insects14020175

**Published:** 2023-02-10

**Authors:** Rosan Adhikari, Dakshina R. Seal, Bruce Schaffer, Oscar E. Liburd, Rafia A. Khan

**Affiliations:** 1 Tropical Research and Education Center, University of Florida, Homestead, FL 33031, USA; 2 Entomology and Nematology Department, University of Florida, Gainesville, FL 32611, USA

**Keywords:** Asian bean thrips, melon thrips, distribution pattern, optimum sample size

## Abstract

**Simple Summary:**

Melon thrips and Asian bean thrips are the major insect pests of beans, inflicting economic losses on bean growers. Thrips infestations impose direct damage by feeding on leaves, flowers and fruits and indirect damage by transmitting viral diseases. Bean growers control thrips pests by spraying conventional insecticides without having basic information about their behavior and biology. This kind of nonjudicial use of insecticides causes the deterioration of the control program by enhancing pest populations. The present study was conducted in six bean fields with variable environmental conditions. Asian bean thrips occur mostly on flowers, and melon thrips are found on host foliage. Management decisions should be implemented based on evaluating population density in flowers and leaves. The distributions of Asian bean thrips, melon thrips and their larvae were mostly aggregated. However, to determine their population density, the number of samples should be collected at a 0.10 or 0.20 precision level based on a predetermined density of 5–10 adults per five-leaf sample. This is a cost-effective and accurate sampling method.

**Abstract:**

Asian bean thrips, *Megalurothrips usitatus* Bagnall, are a serious pest of vegetable crops, especially leguminous crops, across the Asian continent. In Florida, it is a new invasive pest of snap beans. In 2019, it was recorded for the first time in the United States in snap bean (*Phaseolus vulgaris*) fields. Another thrips species, melon thrips, *Thrips palmi* Karny, is also a serious pest that affects several vegetable crops. Within-plant and within-field distribution patterns of *M. usitatus* and *T. palmi* were determined in snap bean fields in southern Florida. The highest number of both thrips species (Asian bean thrips and melon thrips) in snap beans were in flowers, followed by leaves and pods. Both adults and immatures of these thrips exhibited regular to clumped distribution patterns in bean fields. Several statistical indices showed agreement in the distribution patterns of Asian bean thrips, melon thrips, and larvae, irrespective of sampling units and plot size, in three years of study. In most instances, the distribution of Asian bean thrips and melon thrips was aggregated. This study assessed the optimum sample size to accurately determine the population density of these thrips for management purposes. The results from this study will be useful for implementing targeted management programs against thrips pests, thereby reducing labor costs and time. This information will also help reduce agrochemical use.

## 1. Introduction

Florida is the major snap bean (*Phaseolus vulgaris* L.)-producing state in the United States. During the winter season, almost 100% of the country’s fresh snap beans are grown in Florida. The southeastern region of Florida, mainly Miami-Dade County, is the major (57%) snap bean-producing region in the state [1]. Snap bean growers in this area of Florida face severe losses from many native and invasive insect pests. Among insect pests, thrips as a group cause significant damage and can disperse and exist in a wide range of habitats; tropical and subtropical regions are suitable for the survival and colonization of thrips [2].

Asian bean thrips, *Megalurothrips usitatus*, are a newly arrived invasive species, first recorded in Miami-Dade County, Florida, in 2020 [3]. This is the first record of *M. usitatus* in the continental USA. The common hosts are various leguminous crops with a preference for snap beans. Damage is mainly caused by the direct feeding of larvae on leaves, flowers, buds, and pods. A severe infestation can cause 100% crop loss (D.R. Seal, personal observations). *Thrips palmi* Karny (Thysanoptera: Thripidae) is another important pest of beans and other vegetable crops, causing similar damage as *M. usitatus*. Both *M. usitatus* and *T. palmi* are native to Asia.

In the past, various researchers often had difficulties identifying thrips to species [4]. Due to multiple biotic (i.e., availability of food sources) and abiotic factors (climatic conditions), thrips have a diverse host range, and sometimes host ranges overlap among thrips species [5,6,7]. Some researchers reported that the *T. palmi* population in southern Florida was higher in the fall and spring than in summer due to unfavorable climatic conditions for thrips reproduction during the summer [8]. Adverse climatic conditions for reproduction include high summer temperatures and frequent precipitation. Information about within-plant distribution and population dynamics of thrips will provide information for locating pest infestations early in the season.

Insect distribution is positively correlated with environmental factors. In general, an insect’s population can be distributed in three ways: random, clumped, or regular [9]. The distribution of insects often depends upon the population density in a field. When sampling, a low number of insects results in a low capture rate, which indicates a random distribution of insects [10]. In a random distribution pattern, every insect has an equal chance of occurring in an area of the habitat. The denser the pest population, the more aggregated the distribution in the field, resulting in a clumped pattern. If an individual insect’s presence at one point increases the probability of another individual being nearby, then the distribution is clumped [9]. On the contrary, if an individual’s presence at one point decreases the likelihood of another being nearby, then the pattern is more uniform or regular. The distribution pattern of melon thrips (*T. palmi*) in snap bean and chilli thrips (*Scirtothrips dorsalis*, Thysanoptera: Thripidae) in pepper were aggregated in the field [11,12]. Detecting such aggregation at any point of the crop growing period may be helpful in addressing pest problems selectively, thereby reducing the cost of pest control. For example, common blossom thrips’ density is high at the edge of a tomato field at the beginning of an infestation. However, regardless of the distribution pattern, insecticide application is uniform in the field [13]. Such an application of insecticides can cause environmental, economic, and ecological damage. In addition to within-field distribution, thrips exhibit a preference for specific plant parts. Thrips such as *Frankliniella schultzei* Trybom (Thysanoptera: Thripidae) and *Frankliniella fusca* (Hinds) (Thysanoptera: Thripidae) mostly feed on flowers; however, their feeding preferences are not limited to flowers only. Studies showed that chilli thrips were most abundant on the top young leaves of pepper plants, followed by middle and lower leaves [12]. Other researchers reported that larvae of *F. schultzei* feed on the leaf buds of apples, whereas adults feed on leaves as well as flowers [14]. Overall, information about the distribution pattern and feeding behavior of thrips is essential for developing an effective management program.

The objective of this study was to determine the within-field and within-plant dispersion pattern of Asian bean thrips (*M. usitatus)* and melon thrips (*T. palmi*) in snap bean fields in southern Florida. These two thrips species were common in south Florida bean fields during this study. The main emphasis of this study was Asian bean thrips because it is a recently arrived invasive pest of beans.

## 2. Materials and Methods

### 2.1. Study Area and Crop Management

The within-field distribution of thrips was determined in six commercial snap bean fields in Homestead, Florida, USA: two fields in 2020 (Field 1 and Field 2), two fields in 2021 (Field 3 and Field 4), and two fields in 2022 (Field 5 and Field 6). The profile of each field (location, size, and a brief description of the ecology) is shown in Table 1. The within-plant distribution study was conducted in Field 1 and Field 2 in 2020 and repeated in Field 3 in 2021.

The soil type in each field is Krome gravelly loam or Chekika sandy loam [15]. All fields were planted and maintained following standard commercial practices, including planting, fertilizer applications, irrigation, and crop management, as described in the Vegetable Production Handbook of Florida [16]. Raised open-soil beds (0.91 m wide, 20.3 cm high) were prepared with a Kennco superbedder (Kenco Manufacturing Company Inc., Atoka, OK, USA) to plant beans. Beds were spaced 60.1 cm apart, with one row of bean plants in the center of each bed. Plants in each row were spaced 5.1 cm apart. Plants were grown by direct seeding of Caprice green bean seeds, which were placed individually into a 2.54-cm-deep hole created by a seed planter (3-Point Crop-Seeder, Zoro #: G204039667, China). Immediately prior to seeding, 953 kg/ha of 7-11-14 (N-P-K) fertilizer was incorporated into the soil. The second application of granular fertilizer 8-8-16 (N-P-K) at 953 kg/ha was applied as a side dressing 25 days after seeding. Plants were irrigated weekly during the growing period with a water cannon by applying 2.54 cm of water each time.

### 2.2. Sampling for Within-Plant Distribution of Thrips

This study was conducted in Field 1, Field 2, and Field 3. The first two fields were sampled in 2020, and the third field (Field 3) was sampled in 2021. From each field, four 6-m long by 0.92-m.wide plots were randomly selected to sample for within-plant thrips distribution. From each plot, 10 leaves, 10 flowers, and 10 bean pods were collected from 10 randomly selected plants. The samples were placed in a 1-L plastic cup, with each plant part in a separate cup to assess the number of thrips on each part of the plant. Each sample cup was covered with a lid to prevent thrips from escaping. All samples were transported to the Insect IPM laboratory at the Tropical Research and Education Center, UF-IFAS, Homestead, Florida, USA. Samples were soaked in 70% ethanol for 30 min to dislodge the various life stages of thrips. Plant parts were carefully removed from the cup, leaving thrips in ethanol. The contents in ethanol were sieved through a 25-μm sieve (USA Standard Testing Sieve, W. S. Tyler, OH, USA) [17]. The residue in the sieve was transferred into a Petri dish (10 cm diameter), and the numbers of adults and larvae of various thrips species were counted using a compound microscope (Leica wild M3Z, Micro-optics of Florida Inc., Plantation, FL, USA) at 12× magnification. Asian bean thrips and melon thrips were separated [18] based on color (Asian bean thrips are dark and melon thrips are yellowish), antennal segment (Asian bean thrips have 8 and melon thrips have 7), fore-wing (Asian bean thrips have a banded forewing and melon thrips have no bands), and the setal comb on the posterior margin of the eighth abdominal tergite (in Asian bean thrips, it is incomplete, and in melon thrips, it is complete) [19,20]. The larvae were not identified due to the lack of any reliable identifying key. As Asian bean thrips are a newly introduced pest with unknown biology and behavior, we will address various aspects of its males and females. In the instance of melon thrips, we will discuss it.

### 2.3. Sampling Thrips for Within-Field Distribution

Each field was divided into four equal quadrants of 267 m^2^. From each quadrant, 48 plots were randomly selected, where each plot was 5.57 m^2^. Each quadrant was considered a replicate. Thus, there were four replicates in each field.

From each plot, 10 fully expanded young leaves and 10 flowers (composite flowers consisting of 5–10 florets, pods or fruit) from ten randomly selected plants per plot were collected and placed in a 1-L plastic cup, with each plant part in a separate cup to determine the number of thrips on each part of the plant. The rest of the methods for processing samples and identifying thrips were described above for the within-plant distribution study. The within-field distribution was studied by using areas of two sizes, 11.14 and 44.59 m^2^, combining 2 and 8 plots together. Leaf and flower samples were collected accordingly.

### 2.4. Statistical Analysis

#### 2.4.1. Within-Plant Distribution

For the within-plant distribution study, data were square-root transformed before analyses. Non-transformed means are reported in the tables. Transformed data of the within-plant distribution of thrips were analyzed using a mixed model ANOVA with the fixed effects consisting of plant parts and random effects consisting of blocks in a randomized complete block design (PROC GLIMMIX model [21]). In the PROC GLIMMIX model, the method of Kenward–Roger was used to estimate the degrees of freedom. Means were separated using Tukey’s honestly significant difference (HSD) test. All data were analyzed at the 5% level of significance using SAS statistical software (SAS Institute, Cary, NC, USA).

#### 2.4.2. Within-Field Distribution

It has been suggested by several researchers that when studying insect distributions, multiple indices should be used because there is no single index that perfectly satisfies all aggregation studies [22,23,24,25]. Therefore, we used six statistical indices in this study: Taylor’s power law, Iwao’s patchiness regression, index of dispersion, Green’s index, mean crowding, and Lloyd’s index of patchiness (also called Lloyd’s mean crowding). Taylor’s power law [26] and Iwao’s patchiness regression [27] models were used to calculate the dispersion of insects. Taylor’s power law (b) and Iwao’s patchiness regression (β) provide information about the type of dispersion, which is based on the slope value of the models. When the slope value (b and β) is significantly (*p* < 0.05) >1.0 or <1.0, the distribution is considered aggregated or uniform (regular), respectively. Otherwise, the distribution is considered random (b or β value not significantly different from 1.0). The b and β values were calculated using the general linear regression model in SAS [10,21].

b = (logs² − loga)/logx^−^------------(1, Taylor’s power law)

[(s²/^x^ x^−^) − 1] + ^xx^ x^−^ − α----------------(2, Iwao’s patchiness regression)

We also used the index of dispersion to determine the distribution pattern of count data, which is indicated by the value of the variance-to-mean ratio (VMR) [28]. When the value of VMR is >1.0, the distribution of the experimental population is considered clumped; a VMR < 1.0 indicates a regular distribution, and a VMR = 1.0 indicates a random spatial distribution. The index (I) is estimated by the equation:

$$I=\frac{s^{2}}{\hat{m}}=\frac{\sum_{i=1}^{n}{\left(x_{1}-\hat{m}\right)}^{2}}{\hat{m}\left(n-1\right)}$$

where:

*s*^2^ = sample variance,
$\hat{m}$ = sample mean,
*x*_1_ = number of thrips found in the sample units,
*n* = number of sample units.

Green’s index or Green’s coefficient (C_x_) was calculated using the equation:

C_x_ = (σ^2^/m − 1)/(Σx − 1).

The value of the index ranges from −1.0 to 1.0, where a negative value indicates a uniform distribution; 0 indicates a random distribution, and 0.1 to 1 indicates aggregation [25].

Mean crowding (m_x_) was estimated by the equation: m_x_ = mean + (variance/mean) 1.0.

Lloyd’s index of patchiness or Lloyd’s mean crowding defines the mean number per individual of one species in relation to other species in a defined area [29]. An index >1.0, indicates aggregation; an index = 1.0 indicates a random distribution, and an index < 1.0 indicates a uniform or regular distribution.

The percent aggregation was calculated to generalize the pattern for a response variable. Percent aggregation was determined by adding all aggregation responses under that variable and dividing it by the sum of all responses, including aggregation, random and regular, and multiplying the outcome by 100 (no. of aggregation response/sum of all responses) × 100 = percent aggregation for a specific variable). Response variables were thrips species and larvae. The effects of year, field, plot size, linear regression model, and sample type (leaf or flower) were assessed for the response variables and expressed as a percentage of the total.

The following equation was used to determine the optimum sample size, which was developed by Wilson and Room [30]:

N = c^2^tax^b−2^

where c is the reliability (half of the width of the confidence interval as a percentage of the mean), a and b are the intercept and slope values derived from Taylor’s power law or Iwao’s patchiness regression, x is the theoretical mean density, and t is Student’s t value determined with n − 1 degrees of freedom. In the present study, the sample size was determined at three levels of precision (0.10, 0.20, and 0.40) for predetermined densities of 5 and 10 adult male Asian bean thrips, female Asian bean thrips, melon thrips, and larvae in 10 flower or leaf samples per 11.14 m^2^ section.

## 3. Results

### 3.1. Within-Plant Distribution

In 2020, the mean number of male Asian bean thrips was significantly higher in flowers (mean= 41. 70) than in leaves (mean = 2.62) and pods (mean = 0.08) in Field 1 and Field 2 (flowers = 10.29, leaves = 0.58, and pods = 0.04), as well as in 2021 in Field 3 (flowers = 5.25, leaves = 0.70, and pods = 0.10) (Table 2). When total thrips populations (Asian bean thrips males + Asian bean thrips females + melon thrips + larvae) were considered, the number of thrips was much higher in 2020 in Field 1 (mean = 229.68) than in 2020 in Field 2 (mean = 80.43) and 2021 in Field 3 (mean = 30.39) based on flower samples (Table 2).

The female Asian bean thrips population in flowers (mean = 74.85) was two and five times higher in Field 1 (2020) than in Field 2 (2020) and Field 3 (2021), respectively. The number of female thrips in flowers was 37 and 115 times higher than in leaves and pods, respectively (Table 2, Field 1). Melon thrips abundance per sample was significantly higher in flowers than in leaves and pods, except in Field 3.

### 3.2. Within-Field Distribution Based on Flower and Leaf Samples

**Figure 1:**
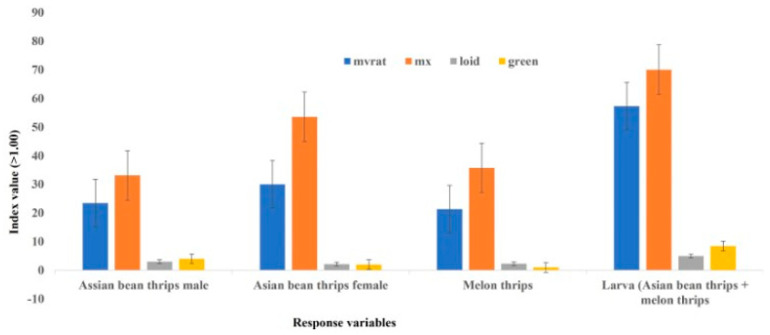
Determination of distribution pattern of thrips using mvratio, index of disperson (mx), Lloid’s index of patchiness and Green’s index.

In 2020 in Field 1, the slope values (b) for Asian bean thrips males were significantly higher than 1.0 in small plots (11.14 m^2^) and large plots (44.59 m^2^), irrespective of the model used, indicating an aggregated distribution (Table 3). In Field 2, male distribution was aggregated in small plots when Taylor’s power law was used; otherwise, males were distributed in random and regular patterns irrespective of the linear models used and plot sizes. In Field 3, the distribution of males was regular according to Taylor’s power law; otherwise, it was aggregated irrespective of the linear model used and plot size. In Field 4, males were distributed in a regular pattern, irrespective of the model and plot size. In Field 5, the distribution of Asian bean thrips males mirrored Field 4. However, in Field 6, males were distributed in a regular pattern in the small plots and in an aggregated pattern in large plots, irrespective of the linear regression model used. When all fields were combined, Asian bean thrips males showed an aggregated pattern of distribution irrespective of the linear regression models used (Table 3). Other statistical indices, including the index of dispersion, mean crowding, Green’s index, and Lloyd’s mean crowding, also showed an aggregated distribution of Asian bean thrips males (Figure 1).

Asian bean thrips females’ distribution pattern was variable based on individual fields (Table 4). In Field 1, the distribution of females was regular and random for Taylor’s power law and Iwao’s patchiness regression, respectively, in small plots. However, in the large plots, the female distribution was aggregated, irrespective of the linear regression model used. In Fields 2 and 3, females were distributed in an aggregated pattern, irrespective of plot size and the linear regression model used. The distribution pattern of females in Field 4 was regular for Taylor’s power law and random for Iwao’s patchiness regression, irrespective of plot size. In Field 5, the distribution of females in the small plots was similar to Field 4 using both models; however, in the large plots, females’ distribution was aggregated using Taylor’s power law and random using Iwao’s patchiness regression. In Field 6, Asian bean thrips females in small plots followed a similar distribution pattern (regular for Taylor’s power law and random for Iwao’s patchiness regression), as in Field 4. The distribution of females in the large plots of the same field was aggregated for Taylor’s power law and random for Iwao’s patchiness regression. When all fields were combined, Asian bean thrips females showed an aggregated distribution pattern in small and large plots, irrespective of the linear regression model used (Table 4). Based on the combined data from all fields, the index of dispersion, mean crowding, Green’s index, and Lloyd’s mean crowding also indicated an aggregated distribution of Asian bean thrips females (Figure 1).

Melon thrips’ distribution was aggregated in a small plot in Field 2 according to Taylor’s power law; otherwise, the distribution was regular in other fields (Table 5). Using Iwao’s patchiness regression, the distribution was aggregated in Field 2; otherwise, it was regular in Fields 1 and 4 and random in Fields 3, 5 and 6. In the large plots, the melon thrips’ distribution was aggregated in all fields except in Fields 4 and 5, where the distribution was regular using Taylor’s power law and random in Field 5 using Iwao’s patchiness regression. When all fields were combined, the distribution of melon thrips was aggregated, irrespective of the linear regression model used, plot size (Table 5), and other statistical indices (index of dispersion, mean crowding, Green’s index, and Lloyd’s mean crowding) (Figure 1).

Larvae populations in the bean fields predominantly consisted of both Asian bean thrips and melon thrips. Larval populations in small plots were distributed either in a regular pattern or an aggregated pattern (Table 6). Larvae were regularly distributed in Fields 1, 4, 5 and 6 and aggregated in Fields 2 and 3, irrespective of the model used. When large plots were considered, larvae were aggregated in Fields 2 and 3, irrespective of the model used. Otherwise, the distribution of larvae was random in Field 1 and regular in Fields 3, 4 and 5 according to Taylor’s power law; they were regular in Fields 1, 4, 5 and 6 according to Iwao’s patchiness regression. However, when all fields were combined, larvae distribution was aggregated irrespective of the model used and plot size, except in Field 6 with a regular distribution pattern in large plots using Iwao’s patchiness regression (Table 6). The index of dispersion, mean crowding, Green’s index, and Lloyd’s mean crowding indicated an aggregated distribution of larvae when data from all fields were combined (Figure 1).

**Figure 2:**
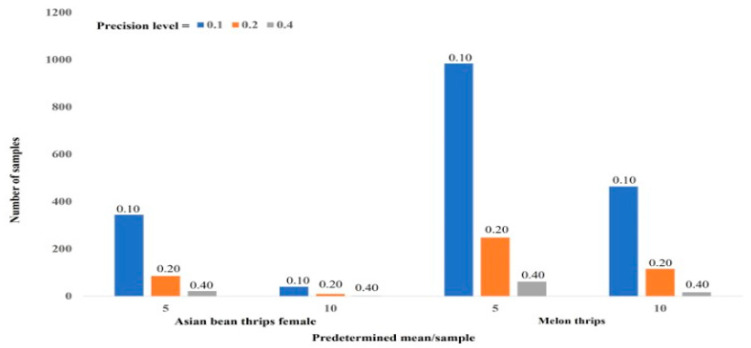
Determination of sample size by using three levels of precision at predetermined densities of 5 and 10 thrips/leaf sample.

We determined the sample size in a field required to understand population abundance using two predetermined means/sample (mean = 5 or 10) at three precision levels. Using a predetermined mean density of female Asian bean thrips adults (5.0 per five-flower sample), 344 samples were required to estimate the thrips’ population density with 0.10 precision (*p* = 0.1), whereas the sample number was only 22 using the same density (5.0 adults per sample) when the precision level was changed to 0.40. Similarly, with a 0.40 precision level using 10 adults per sample, only 11 samples were needed in an 11.14 m^2^ area (Figure 2). Similarly, in the case of a predetermined larvae density of 5.0 per sample at a 0.10 precision level, 1736 samples per 11.14 m^2^ area were required, whereas 820 samples were needed for 0.10 precision using 10 larvae per sample. Similar results were observed for the optimum sample size for male Asian bean thrips and melon thrips.

## 4. Discussion

Asian bean thrips invaded Miami-Dade County, Florida, USA, in 2019. In 2020, about 40% of the bean crops in the area were infested, and the pest had dispersed to additional counties (Miami-Dade Agri-Council, personal communication). In 2021, there was a 2- to 10-fold increase in the abundance and density of thrips, and the pest was observed in almost 100% of bean crops, comprising more than 8000 hectares in Miami-Dade County (D.R. Seal, personal observations). Melon thrips arrived in southern Florida in 1990 and infested all vegetable crops in the area. Snap beans are a suitable host of Asian bean thrips and melon thrips, as documented in the present study. In the within-plant distribution study, the mean numbers of thrips collected in flower samples were significantly higher than in the other plant parts (leaves and pods) in both years of the study in all fields (Fields 1, 2, and 3). In 2021 in Field 3, melon thrips and larval thrips populations were higher on leaves than on flowers. Kawai [31] reported that adult melon thrips were usually found on young leaves of cucumber plants. In sweet pepper, Kawai [31] found more melon thrips adults on flowers and more larvae on fruit than on other plant parts. The difference in within-plant distribution among crops resulted in diverse types of injury. For example, in cucumbers, leaf injuries were common, whereas fruit injuries were common in sweet pepper.

The combined larval population of Asian bean thrips and melon thrips was higher than the adult population in Field 1 in 2020 and Field 3 in 2021. Comparable results were observed by Seal and Stansly [11] in snap beans, where more larvae than adults and more female adults than male melon adults were observed. They also concluded that the infestation of thrips began on the lower leaves and slowly moved to the middle and upper leaves. After depleting food resources in leaves, the melon thrips population moved to the flowers and fruit of snap beans. Irrespective of the field, the thrips population was higher on the abaxial than the adaxial surface of the leaves.

In the present study, the within-field distribution of Asian bean thrips was assessed using traditional methods based on the relationship between the sample mean, *m*, and sample variance, s^2^. Spatial analysis by distance indices (SADIE) and variogram analysis, which were not used in the present study, can provide a true spatial distribution for quantitatively examining changes within the field and should be considered for future studies. In the within-field distribution studies in 2020 (Fields 1 and 2), 2021 (Fields 3 and 4), and 2022 (Fields 5 and 6), Asian bean thrips males showed a variable distribution pattern based on field, plot size and model used. Variability in the aggregation percentage of Asian bean thrips and melon thrips in different fields indicated the dissimilarity of various factors, such as crop phenology, environmental conditions, and the population abundance of thrips in the surrounding vegetation. Similarly, Kakkar et al. [32] reported varied distributions in different fields in the same season, but the reason for this was unclear. In sampling small plots (11.14 m^2^), the aggregation of Asian bean thrips males was observed in two fields using Taylor’s power law and also in a similar number of fields using Iwao’s patchiness regression. The distribution pattern of males was regular in most fields, irrespective of the model used. Increasing the plot size from 11.14 m^2^ to 44.59 m^2^, Asian bean thrips males showed aggregation in a higher number of fields (three for each linear model), irrespective of the model used. The rest of the fields showed a regular distribution pattern. In small plots, *r*^2^ values using Taylor’s power law ranged from 0.0006 to 0.17, whereas *r*^2^ values ranged from 0.81 to 0.98 using Iwao’s patchness regression. A similar pattern of high and consistent *r*^2^ values was recorded for Iwao’s patchiness regression in large plots, indicating a strong fit for Iwao’s patchiness regression model in describing the distribution of Asian bean thrips males.

The distribution of Asian bean thrips females in small plots showed a similar pattern as the male distribution, having an aggregated (Fields 2 and 3) and regular (Fields 1, 4, 5, and 6) distribution in small plots using Taylor’s power law and aggregated (Fields 2 and 3) and random (Fields 1, 4, 5, and 6) distributions using Iwao’s patchiness regression in six different fields. In larger plots, the distribution of Asian bean thrips females was aggregated in 83% of the instances and regular in 17% of the instances using Taylor’s power law. Using Iwao’s patchiness regression, the female distribution was 50% aggregated and 50% random. In commercial fields, we observed that Asian bean thrips infestations were initiated at the edge of a field and spread inward as the season progressed. This dispersion behavior explains the aggregation of Asian bean thrips in larger plots. Our results concur with the findings of Kakkar et al. [32] and Seal et al. [12] for *F. schultzei* and *Scirtothrips dorsalis* (Hood), respectively. Kakkar et al. [33] observed an aggregated distribution pattern of common blossom thrip (*F. schultzei*) adults and larvae in larger plots (1260 m^2^) using Taylor’s power law and Iwao’s patchiness regression models. Similarly, Seal et al. [12] observed an aggregated distribution pattern of *Scirtothrips dorsalis* (Hood) in larger plots of 24 and 48 m^2^, although, in that study, the distribution pattern was regular (1 > b) in smaller plots of 6 and 12 m^2^.

In the present study, the *r*^2^ values in both plot sizes were higher for Iwao’s patchiness regression than Taylor’s power law, supporting a better fit for Iwao’s patchiness regression model than Taylor’s power law in describing the distribution of female thrips. However, the generalized pattern of distribution based on the combined data of all fields was aggregated, irrespective of the model used. This generalized distribution pattern is also supported by other statistical indices (index of dispersion, mean crowding, Green’s index, and Lloyd’s mean crowding), consistently showing an aggregated distribution pattern of Asian bean thrips females (Figure 1).

The melon thrips’ distribution was mostly regular and random in small plots, irrespective of the model used (Table 5). However, in large plots (44.59 m^2^), the distribution was predominantly aggregated irrespective of the model used. Melon thrips are foliage-feeding insects that explore newly emerged young foliage, which is a rich source of nutrients [8]. When the distribution pattern was generalized by combining data from all fields, the melon thrips’ distribution was aggregated, irrespective of plot size and model used. Aggregation in flowers could be under the influence of plant physiology, temperature, the presence of natural enemies, reproduction, and thigmotactic behavior. Milne [33] suggested that aggregation of *F. schultzei* males on any plant part is primarily to attract conspecific females for mating, possibly by the release of sex pheromones.

The distribution of larvae was predominantly regular, irrespective of plot size and model used. However, the generalized pattern of larvae distribution was clumped based on combined data from six fields, irrespective of the plot size and model used. All other statistical indices (index of dispersion, mean crowding, Green’s index, and Lloyd’s mean crowding) were consistent with the linear regression models in describing an aggregated distribution of larvae in bean fields (Figure 1).

The aggregation of thrips species has been established by several researchers. Cho et al. [5] reported an aggregated distribution pattern of adult and immature melon thrips in potato fields. Seal et al. [12] reported a clumped distribution pattern of *Scirtothrips dorsalis* in pepper. A clumped distribution of *F. schultzei* was reported by Kakkar et al. [34] in a cucumber field. Sedaratian et al. [35] reported an aggregated distribution pattern of *Thrips tabaci* in a soybean crop. They also reported that a clumped distribution of *T. tabaci* was probably due to its mode of reproduction. Cho et al. [5] observed a clumped pattern of the spatial distribution of *F. occidentalis* in cucumbers in a greenhouse. Similarly, various studies [36,37,38] showed a clumped distribution pattern of *F. occidentalis* in cucumber, cotton, and strawberries using Taylor’s power law.

Other thrips species, *Aeolothrips intermedius*, *Frankliniella intonsa*, and *Thrips angusticeps*, had aggregated distribution patterns [39]. Larral et al. [40] reported that adults, pupae, and larvae of greenhouse thrips (*Heliothrips haemorrhoidalis* Bouche) in avocado (*Persea americana* Mill.) leaves and fruit had a clumped distribution pattern. In mango (*Mangifera indica* L.) orchards, it was observed that immature specimens of *Thrips hawaiiensis* Morgan, *F. schulzei*, *S. dorsalis*, and *Megalurothrips usitatus* Bagnell were more clumped than adults [41]. Similarly, other researchers noticed a more significant aggregation of immature than adult thrips [5,42,43]. They speculated that the reduced mobility of immature thrips was responsible for this type of clumped pattern. Several factors influence thrips aggregation in the field, including host suitability, fertilizer, irrigation, and climatic conditions [22]. More studies are needed to determine the factors that affect the distribution patterns of Asian bean thrips.

We determined the sample size using a predetermined density of thrips per sample (5.0 and 10.0) at three levels of precision (0.10, 0.20, and 0.40). Southwood [10] suggested 0.25 as the recommended level of precision to assess insect population density. We presume that the suitable precision level to assess male and female Asian bean thrips population density was 0.20. Similarly, a 0.40 precision level was suitable for the density determination of melon thrips and larvae of both species together. If a grower can determine the average density per sample based on previous experience, he or she will be able to collect a smaller number of samples to estimate the correct population density of the pest in the field. As this is a knowledge-based program, growers should have “hands-on” basic training to properly implement a sample size determination program. This information will help in the development of sustainable management practices for Asian bean thrips and melon thrips, two potentially devastating pest species of snap bean in south Florida.

## 5. Conclusions

The densities of Asian bean thrips and melon thrips adults and larvae were high in snap bean flower samples, followed by leaves and pods. All response variables (for Asian bean thrips, melon thrips, and larvae) exhibited variable distribution patterns based on species, sex, size of the sampling area, and the regression model used to assess the aggregation pattern. Based on flower samples in the present study, distribution statistics across all fields indicated an aggregated distribution pattern for all response variables. However, the population distribution should be addressed on a local basis to avoid any failure in developing pest management decisions.

## Figures and Tables

**Table 1:** Profiles of six snap bean fields used for assessing within-field distribution of thrips.

Year	Field No.	Coordinates	Area (m^2^)	East	West	North	South
2020	1	25°30′17.5″ N 80°32′01.3″ W	20,234	Squash, bean and fallow	Bean, housing apartments	Nursery, tomato and bean	Fallow, corn squash
2020	2	25°29′39.1” N 80°30′08.5″ W	28,328	Tomato	Nursery	Bean	Bean
2021	3	25°29′22.9″ N 80°30′13.6″ W	32,375	Squash, bean	Bean, fallow	Tomato, housing	Bean, nursery, and housing
2021	4	25°30′17″ N 80°29′56″ W	20,234	Housing, squash and bean	Nursery, bean and squash	Bean, nursery	Tomato, squash
2022	5	25°30′23″ N 80°28′50″ W	20,234	Squash, okra	Bean, weed, and housing	Squash	Squash
2022	6	25°30′30″ N 80°28′30″ W	24,281	Palm grove	Bean	bean	Fallow

**Table 2:** Mean ± SE number of Asian bean thrips and melon thrips collected from three different fields in different plant parts.

Field	Plant Parts	ABM	ABF	MT	LAR
	Flowers	41.70 ± 2.39 a	74.85 ± 3.36 a	40.65 ± 2.50 a	72.48 ± 3.48 a
Field 1	Leaves	2.62 ± 0.25 b	2.00 ± 0.26 b	4.60 ± 0.57 b	40.08 ± 3.29 b
	Pods	0.08 ± 0.05 c	0.65 ± 0.35 b	0.37 ± 0.19 c	0.29 ± 0.09 c
	Flowers	10.29 ± 0.70 a	35.29 ± 1.70 a	33.60 ±1.88 a	1.25 ± 0.22 a
Field 2	Leaves	0.58 ± 0.14 b	0.20 ± 0.07 b	8.97 ± 0.82 b	0.22 ± 0.08 b
	Pods	0.04 ± 0.04 b	0.08 ± 0.05 b	0.12 ± 0.05 c	0 b
	Flowers	5.25 ±0.58 a	15.04 ± 1.13 a	4.94 ± 0.48 a	5.16 ± 0.62 a
Field 3	Leaves	0.70 ± 0.12 b	1.10 ± 0.19 b	6.18 ± 0.75 a	10.60 ±1.16 a
	Pods	0.10 ± 0.05 b	0.39 ± 0.09 b	0.39 ± 0.19 b	1.00 ±0.42 b

According to the Tukey HSD test, means within the same column followed by the same letter are not significantly different at *p* ≤ 0.05. ABM = male Asian bean thrips, ABF = female Asian bean thrips, MT = melon thrips, LAR = larvae.

**Table 3:** Distribution pattern of Asian bean thrips males in small and large bean plots using flower sample.

11.14 m^2^							44.59 m^2^					
Taylor’s Power Law				Iwao’s Patchiness			Taylor’s Power Law			Iwao’s Patchiness		
Fld1	0.27	−2.75	2.88 ag ^1^	0.91	−2.37	1.12 ag	0.72	−2.05	2.72 ag	0.97	−3.47	1.20 ag
Fld2	0.11	−0.19	1.24 ag	0.76	1.17	0.98 rn	0.28	0.52	0.78 re	0.88	1.77	0.94 re
Fld3	0.05	−0.19	0.87 re	0.78	−0.65	1.13 ag	0.83	−2.32	4.22 ag	0.93	−3.13	1.63 ag
Fld4	0.02	−0.03	0.21 re	0.14	0.86	0.48 re	0.04	0.05	0.30 re	0.54	0.37	0.68 re
Fld5 ^1^	0.02	−0.03	0.21 re	0.14	0.86	0.48 re	0.04	0.05	0.29 re	0.53	0.37	0.68 re
Fld6	0.03	0.04	−0.26 re	0.74	0.33	0.73 re	0.52	−0.74	2.24 ag	0.87	−1.69	1.50 ag
All fields	0.78	−1.06	3.33 ag	0.57	−41.39	3.94 ag	0.9	−1.05	3.33 ag	0.9	−34.40	6.33 ag

^1^ ag = aggregated; rn = random; re = regular.

**Table 4:** Distribution pattern of female Asian bean thrips in various fields based on flower samples.

11.14 m^2^							44.59 m^2^					
Taylor’s Power Law				Iwao’s Patchiness			Taylor’s Power Law			Iwao’s Patchiness		
	*r* ^2^	a	b	*r* ^2^	a	β	*r* ^2^	a	b	*r* ^2^	a	β
Fld1	0.01	1.46	0.27 re ^1^	0.98	2.19	0.99 rn	0.82	12.62	8.07 ag	0.98	−44.80	1.67 ag
Fld2	0.17	−2.96	2.90 ag	0.93	−4.03	1.15 ag	0.61	−5.55	4.84 ag	0.97	−11.53	1.39 ag
Fld3	0.08	−0.26	1.18 ag	0.81	−1.04	1.15 ag	0.19	−0.38	1.54 ag	0.84	0.7	1.04 ag
Fld4	0.03	−0.49	0.70 re	0.94	−0.49	0.95 rn	0.09	−0.52	0.93 re	0.98	−0.75	1.01 rn
Fld5	0.003	0.1	0.26 re	0.93	−0.19	0.96 rn	0.2	−0.96	1.73 ag	0.97	−0.37	0.99 rn
Fld6	0.006	0.36	−0.05 re	0.92	−0.01	0.95 rn	0.2	−0.96	1.74 ag	0.97	−0.37	1.00 rn
All fields	0.63	−1.58	3.18 ag	0.87	−19.47	3.69 ag	0.62	−1.69	3.26 ag	0.66	−55.48	4.41 ag

^1^ ag = aggregated; rn = random; re = regular.

**Table 5:** Distribution pattern of melon thrips in small and large bean plots using flower samples.

11.14 m^2^							44.59 m^2^					
Taylor’s Power Law				Iwao’s Patchiness			Taylor’s Power Law			Iwao’s Patchiness		
Fields	*r* ^2^	a	b	*r* ^2^	a	β	*r* ^2^	a	b	*r* ^2^	a	β
Fld1	0.02	0.77	0.71 re ^1^	0.83	6.99	0.92 re	0.68	−1.47	2.43 ag	0.94	−6.09	1.29 ag
Fld2	0.14	−2.52	2.49 ag	0.95	−1.46	1.07 ag	0.13	−1.19	2.15 ag	0.85	−2.22	1.17 ag
Fld3	0.13	−0.33	0.88 re	0.89	−0.36	1.01 rn	0.74	−1.69	3.38 ag	0.96	−2.51	1.55 ag
Fld4	0.02	−0.09	0.17 re	0.59	0.29	0.68 re	0.14	−0.24	0.83 re	0.81	−0.61	1.10 ag
Fld5	0.008	−0.29	0.37 re	0.84	−0.86	1.04 rn	0.11	−0.35	0.86 re	0.95	−0.63	1.00 rn
Fld6	0.04	−0.34	0.74 re	0.83	−0.06	0.95 rn	0.58	−0.82	1.64 ag	0.99	−0.90	1.07 ag
All fields	0.94	−0.84	2.81 ag	0.91	−16.65	3.37 ag	0.96	−1.39	3.28 ag	0.94	−27.39	4.07 ag

^1^ ag = aggregated; rn = random; re = regular.

**Table 6:** Distribution pattern of thrips larvae in six fields based on flower samples.

11.14 m^2^							44.59 m^2^					
Taylor’s Power Law				Iwao’s Patchiness			Taylor’s Power Law			Iwao’s Patchiness		
Fields	*r* ^2^	a	b	*r* ^2^	a	β	*r* ^2^	a	b	*r* ^2^	a	β
Fld1	0.03	2.86	−0.4 re ^1^	0.67	12.35	0.92 re	0.04	4.59	0.99 rn	0.65	27.03	0.73 reg
Fld2	0.72	0.06	1.15 ag	0.77	−0.50	1.38 ag	0.93	−0.16	2.98 ag	0.96	−1.76	2.62 ag
Fld3	0.12	−0.47	1.33 ag	0.68	−0.73	1.89 ag	0.79	−0.26	1.36 ag	0.97	−0.28	1.05 ag
Fld4	0.01	−0.16	0.14 re	0.36	0.17	0.78 re	0.13	−0.03	0.71 re	0.61	0.01	0.93 re
Fld5	0.04	−0.12	0.37 re	0.6	0.23	0.77 re	0.09	0.001	0.42 re	0.89	−0.06	0.88 re
Fld6	0.01	−0.16	0.14 re	0.37	0.17	0.78 re	0.13	−0.03	0.71 re	0.61	0.01	0.93 re
All fields	0.92	0.17	2.37 ag	0.84	−10.89	5.77 ag	0.79	0.88	1.76 ag	0.57	10.18	4.21 reg

^1^ ag = aggregated; rn = random; re = regular.

## Data Availability

The data presented in this study are available on request from the corresponding author.
